# 

**Published:** 1888-03

**Authors:** 


### THIRD ANNUAL MEETING OF LONE STAR MEDICAL ASSOCIATION.

The President desires the Lone Star Medical Association to convene in Austin on the 14th of May, 1888, instead of the 23d of June, for the following reasons:

1. There is usually not so much illness at that season, and we can better leave our work.

2. It will enable you to be present at the dedication of the State Capitol and the Interstate drill.

3. Last, but not least: Reduced rates over all the railroads, being $5 for round trip from any part of the State.

Please send in at once the subject of thesis. Address:

J. F. McKinley, M. D., President,
Over 907 Congress Avenue, Austin, Texas.

M. A. Majors, M. D., Ass't Sec'y.

